# O.4.6-1 Examining the feasibility, acceptability, and effectiveness of a self-compassion workshop in physical education class for adolescent females

**DOI:** 10.1093/eurpub/ckad133.220

**Published:** 2023-09-11

**Authors:** Vanessa Marie Coulbeck

**Affiliations:** Western University, Canada; vcoulbec@uwo.ca

## Abstract

**Purpose:**

In adolescence, enrollment in Physical Education (PE) drastically drops in the transition from grade 9 to 10 – particularly for females who disengage at higher rates than males after completing provincial PE curricular requirements. This is problematic since engagement in PE has the potential to shape and guide young females’ perceptions of movement, their bodies, and coping with life’s difficulties through physical activity. Body image distress may be one reason to explain PE disengagement therefore, may warrant intervention. Self-compassion is an emotion regulation strategy that helps deal with body-related distress and may be an effective strategy to help females cope with these negative experiences in PE and PA beyond. This is the first pilot study to examine the feasibility, acceptability and effectiveness of a self-compassion psychoeducation intervention in PE.

**Methods:**

A pragmatic one-group pre-test post-test design, with 1-month follow-up was used. The sample consisted of n = 52 students from 2 Canadian high school grade 9 grade 10 females PE classes. The intervention consisted of a 75 minute self-compassion workshop. Feasibility and acceptability were assessed using multiple data sources from a 75-minute focus group, and 1-month follow up survey data. Effectiveness was examined using change in scores on PE attitudes, PA attitudes, self-compassion, and body-image coping.

**Results:**

There was overall feasibility evidence for the intervention, whereby, all available classes and teachers agreed to host the workshop. The majority of students completed the pre-survey 84.6% (44/52), workshop 71.5% (37/52), and focus group session 92.3% (48/52), and expressed interest in having follow-up workshops 60.7% (17/28). The intervention demonstrated acceptability, as indicated by students indicating high scores on workshop acceptability, ethical conduct, effectiveness, limited negative side effects, material being beneficial and presented in a useful way. Small improvements were shown for self-compassion (d = 0.0761), PE attitudes (d = 0.0761) and PA attitudes (d = 0.245) from pre-workshop to follow-up.

**Conclusions:**

The knowledge gained from this pilot study may enhance capacity for delivery of scalable curriculum-informed physical education promotion strategies which may be broadly transferrable to physical activity programs.

